# Complex networks interactions between bioactive compounds and adipose tissue vis-à-vis insulin resistance

**DOI:** 10.3389/fendo.2025.1578552

**Published:** 2025-05-13

**Authors:** María Barrera-Esparza, Elizabeth Carreón-Torres, Angélica Saraí Jiménez-Osorio, Julieta Angel-García, Octavio Jiménez-Garza, Olga Rocío Flores-Chávez, Geu S. Mendoza-Catalán, Diego Estrada-Luna

**Affiliations:** ^1^ Coordinación de Unidades de Segundo Nivel, Oficina Central, Servicios de Salud del Instituto Mexicano del Seguro Social para el Bienestar (IMSS-BIENESTAR), Mexico City, Mexico; ^2^ Physics Institute, Universidad Nacional Autónoma de México (UNAM), Mexico City, Mexico; ^3^ Department of Molecular Biology, Instituto Nacional de Cardiologiía “Ignacio Chaívez”, Mexico City, Mexico; ^4^ Departament of Nursing, Instituto de Ciencias de la Salud, Universidad Autoínoma del Estado Hidalgo (UAEH), Hidalgo, Mexico

**Keywords:** complex networks, adipose tissue, insulin resistance, bioactive compounds, inflammation, oxidative stress

## Abstract

Fatty acids disorders may lead to insulin resistance, resulting in long-term oxidative stress and inflammatory processes, both mediated by adipose tissue. Either in normal condition or obesogenic status, adipose cells components play an important role in several physiological and metabolic conditions. It has been shown that bioactive compounds can influence the development of obesity and its pathological complications such as insulin resistance. In this study, we performed a network between bioactive compounds and adipose tissue vis-a-vis insulin resistance. We constructed a regulatory network of 62 adipocyte cell components that incorporates current evidence of cellular and molecular interactions involved in healthy and obesity states. The network incorporated information about inflammation pathways and inhibition of insulin signaling; insulin signaling and GLUT 4 translocation; triglycerides production; ATP production; M2 macrophages recruitment; adipogenesis and lipolysis as well as mitochondrial biogenesis. Our mathematical model showed a discernment between the impact of various bioactive substances on the transitions from health to obesity and vice versa. We found that anthocyanins, punicalagin, oleanolic acid, and NRG4 proved to be critical nodes in the transition from obesity to the healthy state, due to their switch-on potential to up-regulate the complex network resulting in a beneficial transition.

## 1 Introduction

Obesity is a complex, chronic degenerative disease from multifactorial origin, based on an imbalance in the absorption and use of calories (1) which is usually a risk factor for the development of cardiovascular diseases, some types of cancer, type 2 diabetes, neurodegenerative disorders and liver damage (2–5). Under regular conditions, adipose tissue produces several cytokines and hormones (adiponectin, leptin, resistin, adipokines, chemokines, etc.) which are important in lipid and metabolic homeostasis.

Either directly or indirectly, these biological factors interact with extrinsic and intrinsic factors, which are mainly involved in inflammatory processes, oxidative stress, and lipid profile, mediated adipose tissue. Nevertheless, in a normal condition or inflammation, obesogenic status, adipose cells components and other immune cells (such as M1 and M2 type) play an important role in several physiological and metabolic conditions. These cells exhibit distinct but complementary functions through different pro- or anti-inflammatories mediators such as Nuclear Factor kappa B (NFκ-B) (6), Toll-like receptors family (TLR2 and TLR4, mainly), tumoral necrosis factor-alpha (TNF-α) (7), peroxisome proliferator-activated receptor (PPAR) (8), glucose transporters (GLUT) (9), diverse interleukins (IL) (10), and hormones such as leptin, glucagon-like 1 (GLP1) and adiponectin (11) and Neuregulin 4 (NRG4). Likewise, these factors and obesity per se have been widely described to be directly implicated in the development of insulin resistance, which, in turn, unleashes and exerts diverse other metabolic disorders such as lipid profile dysregulation, mitochondrial dysfunction, lipoperoxidation, etc., leading to diabetes and cardiovascular diseases, mainly (12–17). In recent years, some factors that has been studied with the aim of correcting the abnormalities of glucose resistance include the effect of oxygenation of adipose tissue (18), reducing the levels of C-reactive protein (19), proving the efficacy of the consumption of glucagon-like peptide-1 receptor agonist (20), among others, nevertheless, the results are still heterogeneous. Furthermore, a considerable number of investigations have been performed involving adipose cells components in several biomodels to regulate these changes mentioned above, implementing diets, exercise and physical activity, and bioactive compounds (21–24) among others.

However, the importance of specific dietary composition can affect how food is absorbed and how it is stored, used or dissipated in adipose tissue. In this context, it has been shown that distinct bioactive compounds can influence obesity and its pathological complications such as insulin resistance, inflammation and metabolic syndrome in lipid turnover. Bioactive compounds such as phenolic compounds, triterpenes, anthocyanins, fructooligosaccharides, etc., can directly or indirectly modulate the signaling and expression of genes involved in regulating energy intake, lipid metabolism, adipogenesis in adipose tissue, thermogenesis, lipotoxicity, adipo/cytokine synthesis and inflammation (25–27). Thus, also at the intestinal level, higher concentrations of these molecules can be achieved, modulating the composition, action and function of the microbiome.

Some of this research has been focused on the development of clinical trials, experimental studies on biomodels and cell strains, as well as modelling mathematics to stimulate clinical conditions and to predict physiological patterns using medical physics techniques.

Application of mathematical models to predict biological molecule interactions, tissue behaviors or the natural course of disease (28–30) has provided the basis for comprehension and understanding of disease outcomes, response and progression, being an important field of scientific research for the development of clinical and experimental protocols. Several mathematical models based on systems biology have been previously considered (31–36) to elucidate the mechanisms underlying adipocytes’ homeostasis functions and alterations that drive them to transitions from health to obesogenic state and vice versa. We used a mathematical model based on a regulatory network composed of nodes representing molecular and cellular associations playing important interactions linked to the development of negative physiological pathways in adipose tissue during insulin resistance and obesity. In a first approach, a regulatory network can be characterized by a Boolean analysis, first proposed by Stuart Kauffman in the 60’s as a mathematical model of genetic regulation (37), where all the components only can have two values: zero (completely turned off or inhibited), or one (completely expressed or activated); these values depend on previous states of all nodes in the network. By considering a network formed by n nodes, the state of the node *k* at a time *t+τ* can be described by the mapping $q_{k}\left(t+\tau \right)=f_{k}\;\left(q_{1}\left(t\right),\ldots ,q_{n}\left(t\right)\right)$ ; here, *f_k_* is determined by a logical proposition that satisfies Boole axiomatics. The stationary states of the mapping are determined by the condition $q_{k}\left(t+\tau \right)=q_{k}$ (t), where this can define different cellular phenotypes, defined as obesity or health in this work.

Depending on their value, each component (node) of the network could affect the behavior of some other nodes or even the entire network. The final nodes values determine or can be associated with a physiological state of health or obesity. In a more realistic approach, regulatory networks can be characterized by considering values between zero and one and, once we establish the behavior and connections between nodes through differential equations, we can alter the physiological final state by modifying values of some critical nodes. One of the main objectives of this type of methodology is to identify the critical nodes that have the potential to change the final physiological state when we alter their values and to determine specific molecular factors that may influence different pathways either directly or combined, in order to be used as a predictive-methodological instrument in future experimental studies and clinical trials related to neurological conditions (38, 39).

This study aims to identify by theoretical predictions and mathematical modeling relevant bioactive compounds capable to modulate positive or negative the physiological mechanisms in adipose tissue under insulin resistance to guide experimental work and model biological systems more accurately.

## 2 Materials and methods

In this study, we performed a network between bioactive compounds and adipose tissue vis-a-vis insulin resistance. First, a literature review was performed in metabases including PUBMED, MEDLINE and SCOPUS, considering keywords such as “bioactive compounds” and “adipocyte tissue”, “insulin resistance biomodel” as well as the interaction and effects between them. Articles in English and Spanish were chosen as eligible literature considering randomized clinical and experimental studies published in the last five years. Subsequently, two authors independently reviewed each article in turn to discuss scientific evidence using three steps according to recommendations by Miller (40): a) Identifying the biological pathways linked to the clinical outcomes of the disease, i.e., the mechanism of pathogenesis. b) Distinguishing the dynamics of the bioactive compounds and adipocyte tissue and c) Describing how the dynamics of the bioactive compounds and adipocyte tissue modify the biological pathways leading to insulin resistance.

**Figure 1:**
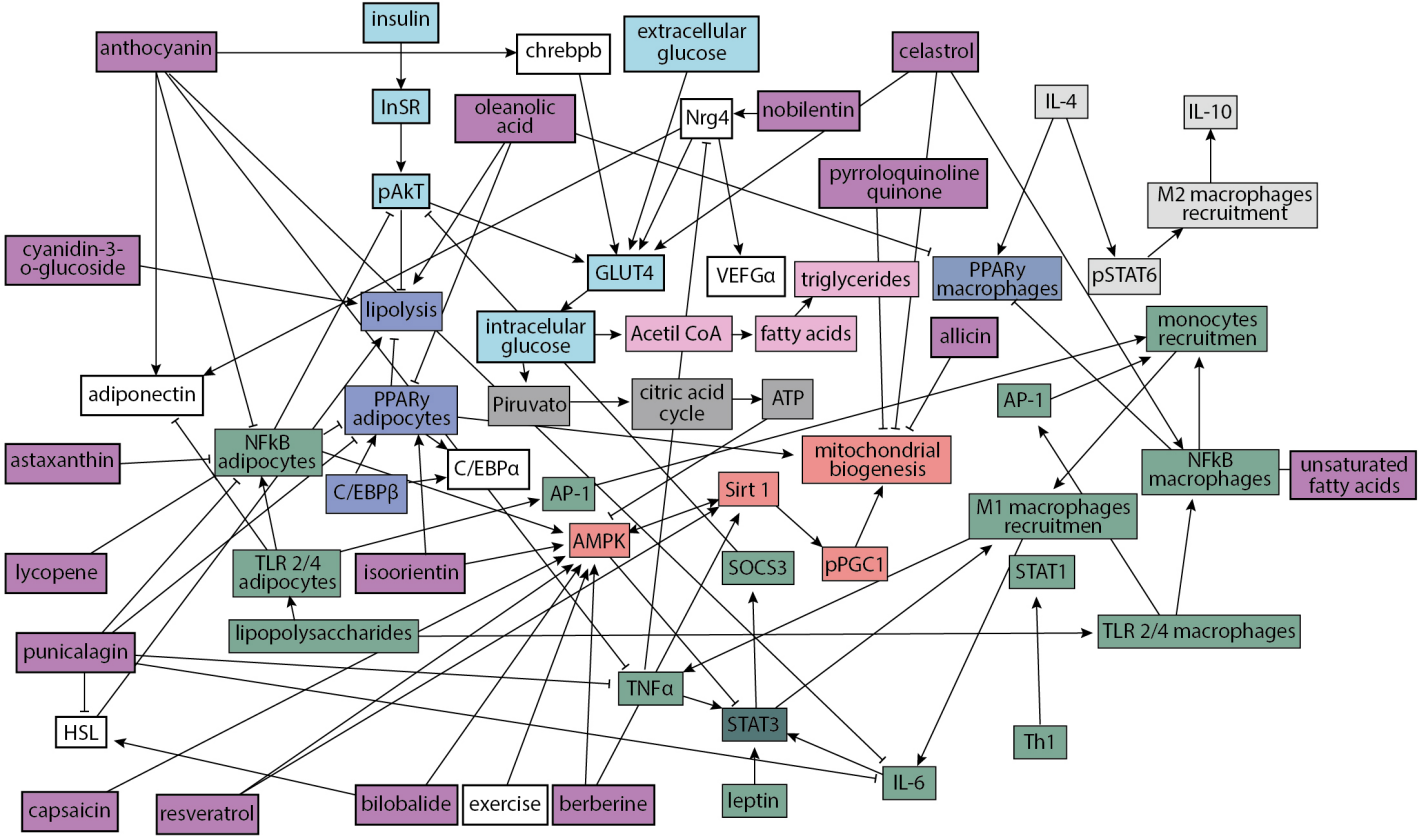
Adipocytes regulatory network. Inflammation pathways and inhibition of insulin signaling (green nodes); insulin signaling and GLUT 4 translocation (turquoise nodes); triglycerides production (pink nodes); ATP production (dark gray nodes); M2 macrophages recruitment (light gray nodes); adipogenesis and lipolysis (navy nodes); mitochondrial biogenesis (red nodes) and bioactive compounds (purple nodes). Interactions with final arrows represent a positive influence of one to another node, a node can “turn on” another node; meanwhile, interactions with red final bars indicate that a node can “turn off” or inhibit another node.

Based on the literature review mentioned above (41–70), we constructed a regulatory network of 62 adipocyte cell components (**Figure 1**) that incorporates current evidence of cellular and molecular interactions involved in healthy and obesity states (**Supplementary Table S1**). The network incorporates information about inflammation pathways and inhibition of insulin signaling; insulin signaling and GLUT 4 translocation; triglycerides production; ATP production; M2 macrophages recruitment; adipogenesis and lipolysis as well as mitochondrial biogenesis; which in presence of adipocyte failure, a condition common in obesity, leads to an increased level of fatty acids in muscle and liver tissue, resulting in a reduction of insulin response in various tissues and bioactive compounds.

In **Supplementary Table S1**, we summarize the information of the literature review; for every node, we associated it with a value between zero and one for a state of health and obesity. Green cells correspond to node’s values greater than red ones (for example, anthocyanin has a higher expression level in health than in obesity). This table represents the attractors of the network: an attractor characterizes a steady state of values that remains stable over time while there are no disturbing external factors that change the final state. These attractors correspond to health and obesity states.

### 2.1 Mathematical description

Each node value can be seen as a variable that may vary from completely turned off (0) to completely expressed (1), and the degree to which an object exhibits a property p is given by a continuous expression µ[p], denoted as characteristic or membership function. The regulatory interactions are embraced by a characteristic function $\mu [w_{k}\left(q_{1}\left(t\right),\;\ldots ,\;q_{n}\left(t\right)\right)]$ . Dynamical properties of the system were subsequently analyzed by means of a system of differential equations (32):


$$\frac{\text{d}{\text{q}}_{\text{k}}}{\text{d}\text{t}}=µ\left[{\text{w}}_{\text{k}}\left({\text{q}}_{1}\left(\text{t}\right),\;\ldots ,\;{\text{q}}_{\text{n}}\left(\text{t}\right)\right)\right]-{\text{α}}_{\text{k}}{\text{q}}_{\text{k}}$$

Where $\mu [w_{k}]$ has a sigmoid structure and *α_k_* is a decay rate.

In this work, we studied the possible phase transitions between health and obesity by considering a set of ordinary differential equations for the expression levels of the network components. The characteristic function may be expressed by means of a function with a sigmoid structure:


$$µ\left[w_{k}\right]=\;\frac{1}{1+exp\left[-b\left(w_{k}\left(q_{1},\;\ldots ,\;q_{n}\right)-w^{thr}\right)\right]}$$

Here, $w^{thr}\;$ is a threshold value that renders $w^{thr}\;$ true (expressed) if $w_{k}>w^{thr}$ . The simplest assumption is to consider $w^{thr}=1/2$ . Parameter b is a saturation rate for the change of the proposition $µ\left[w_{k}\right]$ from an unexpressed to a totally expressed state, being gradual for small b, and steep for large b. In this work we suppose that b=5.5.

For example, for the node insulin receptor, the differential equation that describe the expression level of the node over time is:


$$\frac{dinsr}{dt}=\frac{1}{1+exp\left[-5.5\left(insulin\left(t\right)\right)-0.5\right)]}\;-q_{insr}$$

where $w_{k}=w_{insr}$ *=insulin*, since the node insulin receptor only depends on the insulin node; $w^{thr}=0.5;\;$ *b*=5.5; $\alpha_{insr}$ =1 and $q_{insr}$ is the expression level of the node insulin receptor at the time *t*.

### 2.2 Transitions between states

Dynamical analyses were performed to study the transitions from health to an obesity stage, by employing the continuous regime. This was analyzed with the Wolfram Mathematica computing system, using ordinary equations described in **Supplementary Material 1**. Each attractor in **Supplementary Table S1** was in this step considered as a set of initial conditions of every node, and the analysis allowed to identify cases where the perturbation of a bioactive compound value induced a transition from the original to a different attractor, it was assumed that *α_k_=*1 for every node. This is equivalent to consider, for example, a person who is in an obesity stage characterized by a nutritional deficiency of bioactive compounds’ ingest and we examine if there will be a state transition of obesity to healthy state when the ingest of bioactive compounds increases. In results, the time function is a representation of the dynamic evolution of the nodes, where it is affected by the decay rate *α_k_* and by the saturation rate b. For small values of b, the change of the expression level of a node is gradual, while for large values of b, the node can change in a shorter time. On the other hand, if we increase the decay rate of a node, it will turn off in a short time. We show the time function in the results up to 20, because this is enough to exhibit a stable behavior of a node, where it can be turned on or turned off.

## 3 Results

### 3.1 Transition from obesity to health

**Figure 2:**
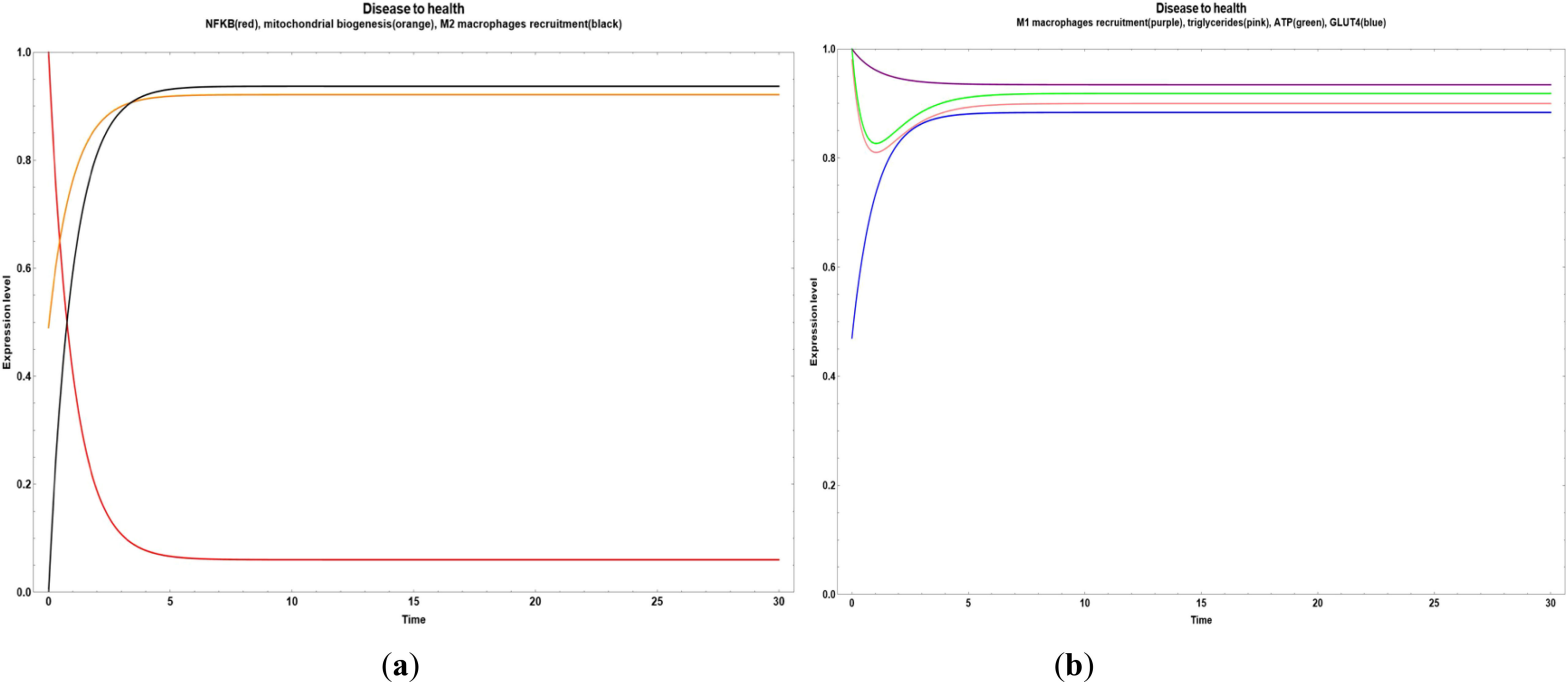
Transitions from disease to an improved health state when all the bioactive compounds are expressed inside of an obesity environment. **(a)** Dynamic transitions related to a decrease of NFκ-B in adipocytes and increase in mitochondrial biogenesis and M2 macrophages recruitment; **(b)** Dynamic transition showing an increase of GLUT 4 expression and a steady state of M1 macrophages recruitment, triglycerides and ATP.

In **Figure 2**, we observe an overall dynamic behavior reliable to a transition from disease (obesity) to health, where the inflammatory values decreased and the M2 macrophages recruitment increased, with a little increase of GLUT 4 expression and a steady state of M1 macrophages recruitment, triglycerides and ATP. This was achieved when all the bioactive compounds transited from small to big values.

#### 3.1.1 Critical nodes: anthocyanin and punicalagin; neuroregulin 4 and oleanolic acid

**Figure 3:**
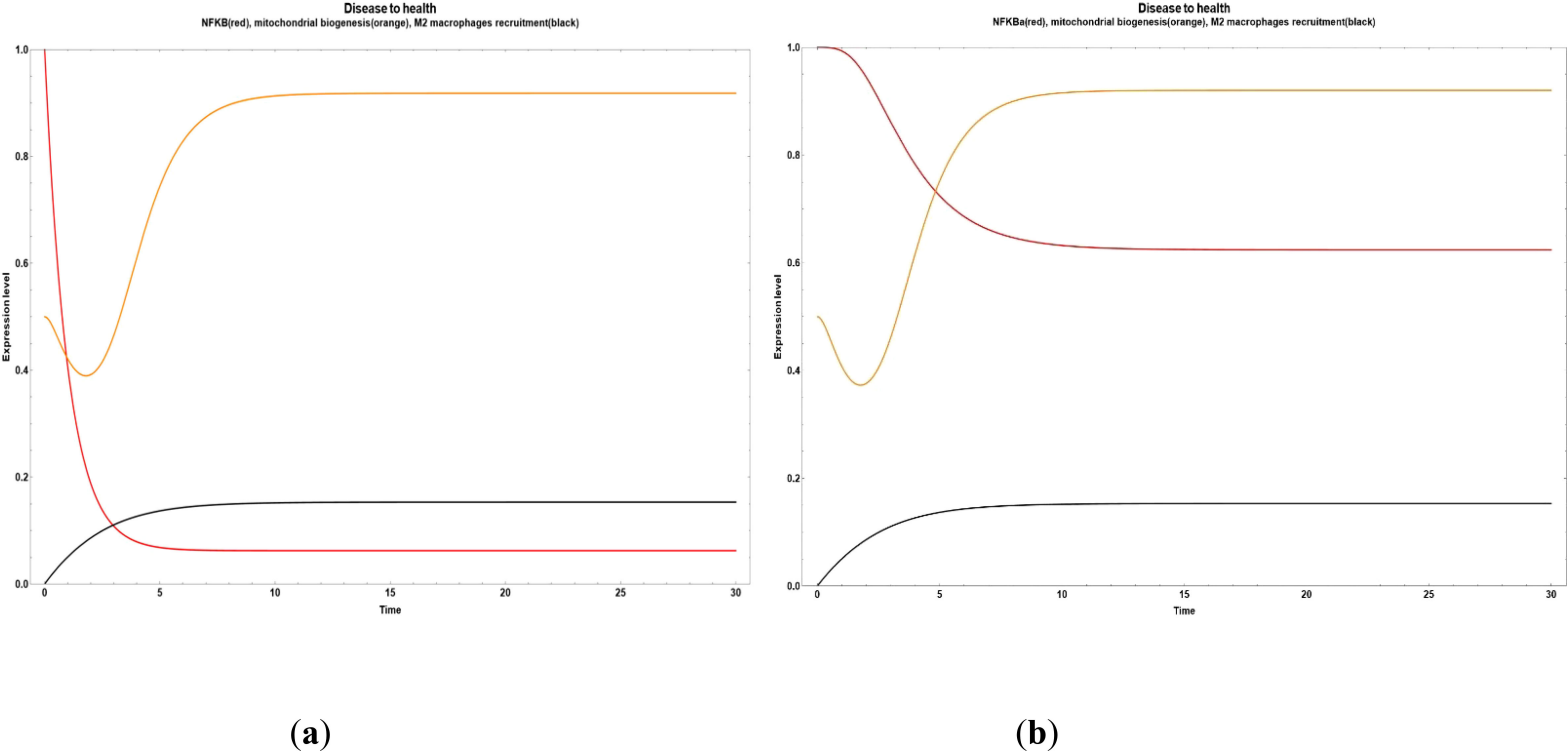
Transitions from disease to an improved health state when some bioactive compounds are expressed inside of an obesity environment. **(a)** Dynamic transitions related to an evident decrease of NFκ-B in adipocytes and a transient increase in mitochondrial biogenesis. In this scenario, we turned on the anthocyanin and punicalagin values **(b)** Dynamic transition showing a decrease of inflammation but not as big as in **(a)**. This evolution is due to the NRG4 increased value.

**Figure 4:**
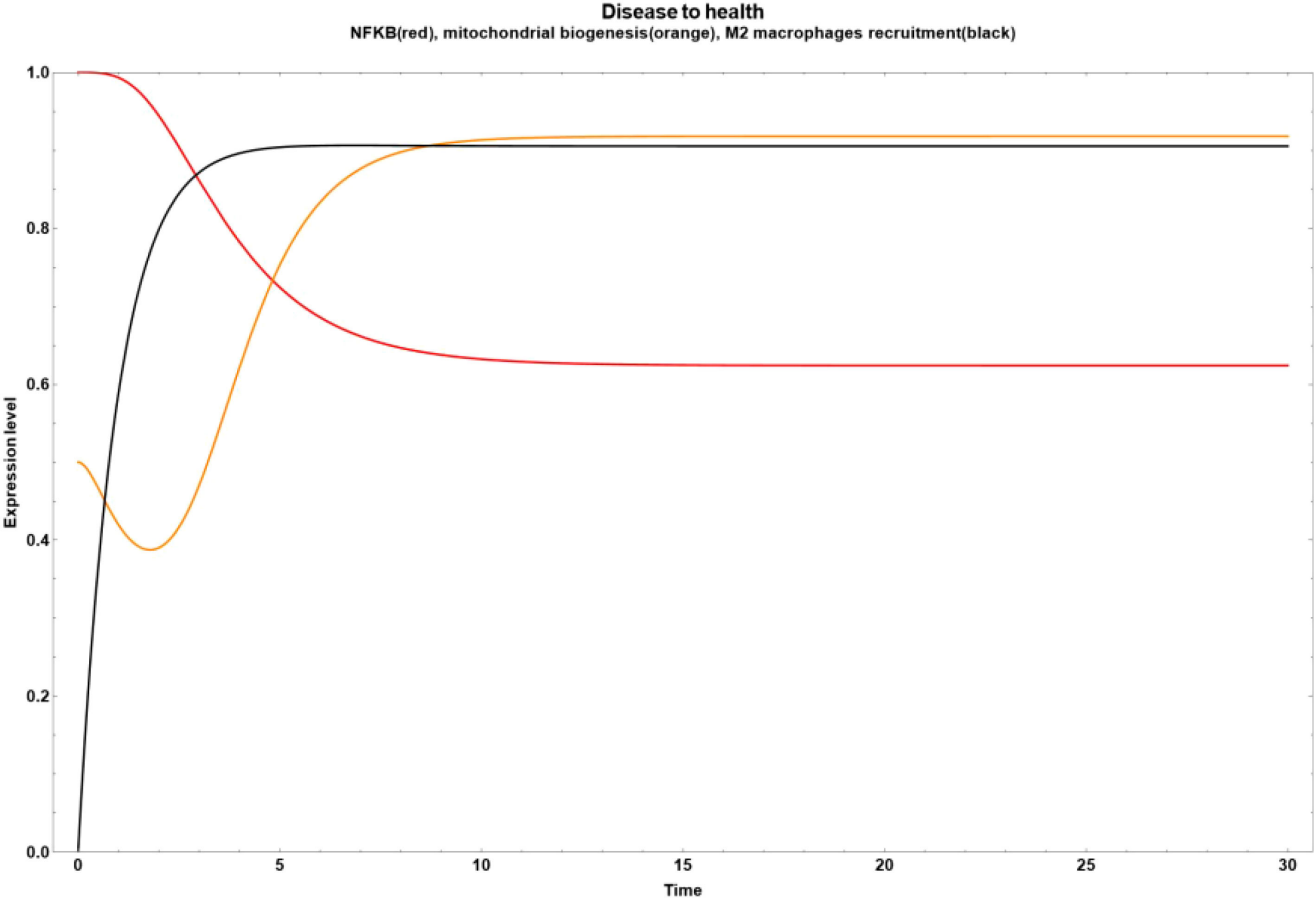
Transition from disease to an improved health state when all the bioactive compounds are turned off, except for oleanolic acid.

We found some critical nodes driving the network final state from obesity to an improved state of health characterized by a reduced inflammatory value. For example, if we increase the anthocyanin and punicalagin values, we observe a drastic reduction in NF-κB (**Figure 3a**). On the other hand, if we increase only the neuroregulin 4 (NRG4) and the oleanolic acid, respectively, the final state transitions to a small reduction in inflammation, compared with the effect of turn on anthocyanin and punicalagin nodes, where they suppressed NF-κB, as it can be seen in **Figures 3b** and **4**. However, the impact of NRG4 on mitochondrial biogenesis is clear, driving an evident increase when the NRG4 is turned on. In **Figure 4**, a marked increase in M2 macrophage recruitment occurred when the value of oleanolic acid is increased.

### 3.2 Transition from health to obesity

**Figure 5:**
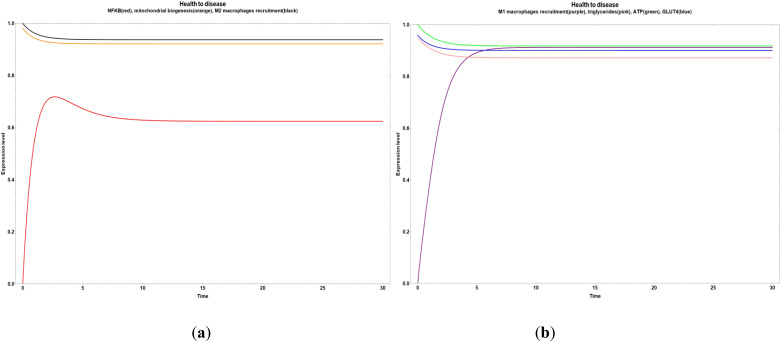
Transitions from health to disease state when all bioactive compounds are sub-expressed. **(a)** Dynamic transitions leading to an overexpression of NFκ-B and a steady expression of mitochondrial biogenesis and M2 macrophages recruitment. **(b)** Dynamic transition from a null to an evident M1 macrophages recruitment and a stable level of triglycerides, ATP and GLUT 4.

In **Figure 5**, we can observe a pathway leading from health to disease with an early increase in NF-κB (**Figure 5a**) and M1 macrophages recruitment (**Figure 5b**). Both cases were stimulated when the values of all the network´s bioactive compounds were reduced.

#### 3.2.1 Critical nodes from health to disease

**Figure 6:**
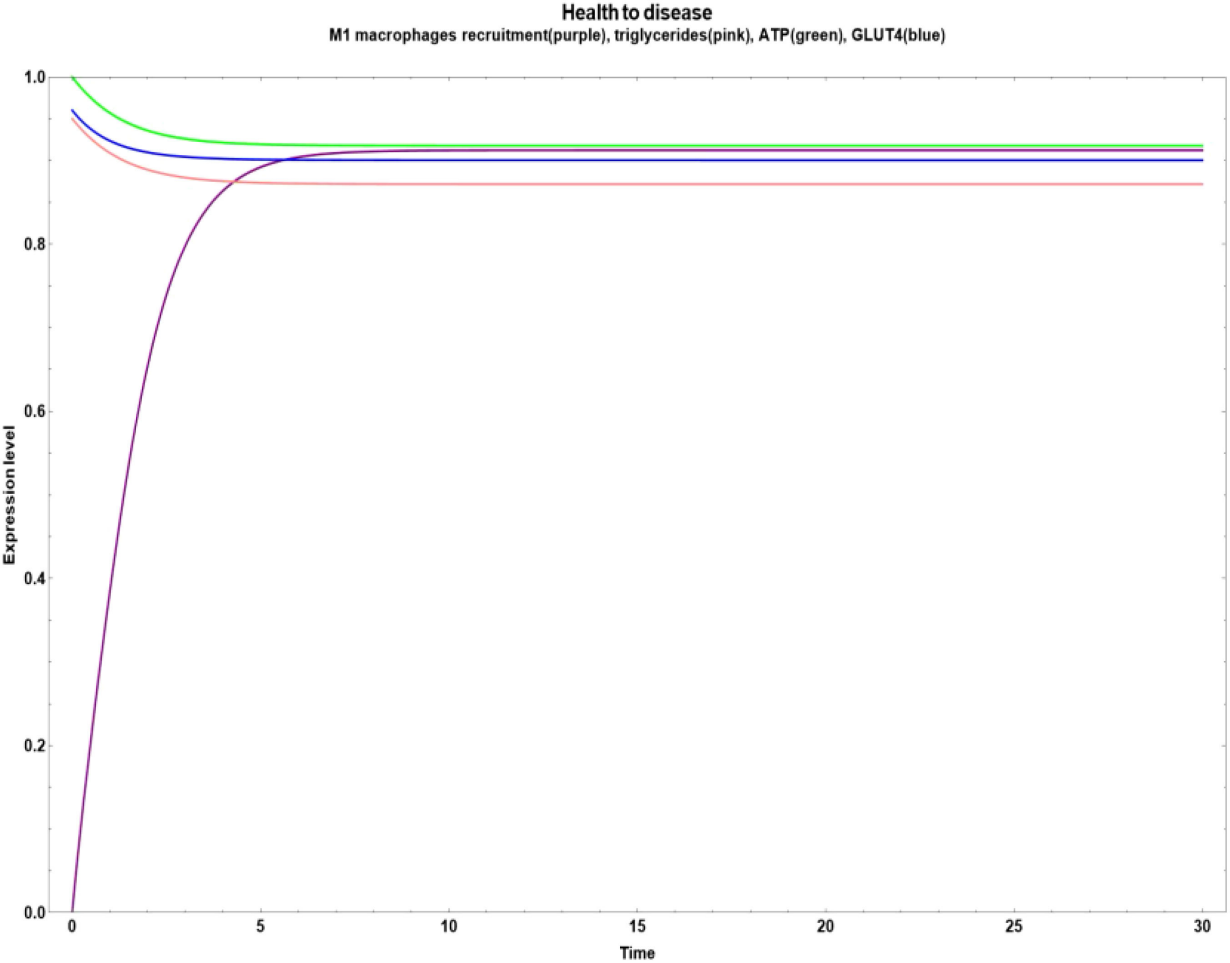
Transition from health to disease characterized by a boost in M1 macrophages recruitment caused by an increase in lipopolysaccharides.

**Figure 7:**
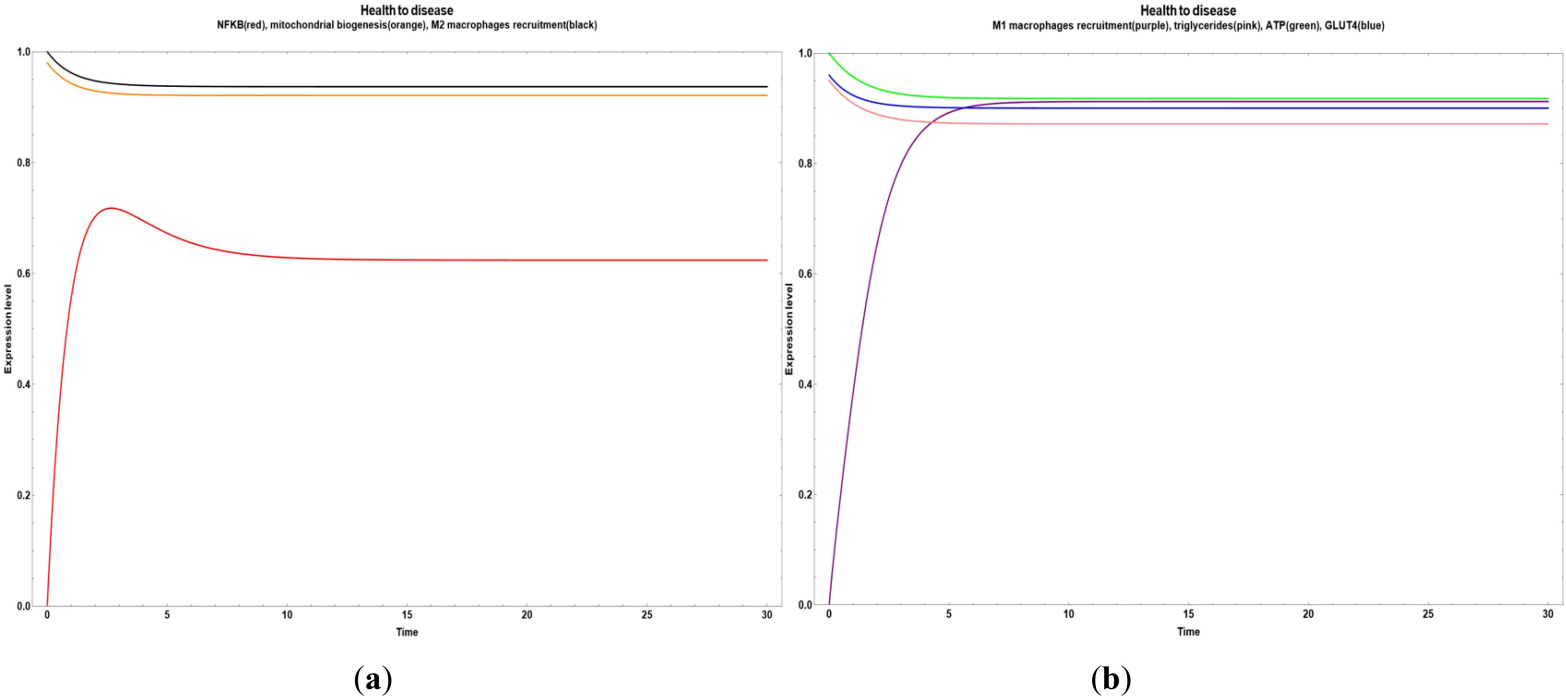
Transitions from health to disease are caused by a decrease in the astaxantin, anthocyanin, lycopene and punicalagin compounds, with a rise in lipopolysaccharides (all at the same time). **(a)** Increase in the NFκ-B inflammatory value. **(b)** Increase in the M1 macrophages recruitment with a constant behavior of triglycerides, ATP and GLUT 4.

We found critical nodes driving the system from health to disease when their values turned on. In **Figure 6**, there is an evident increase in M1 macrophages recruitment when the node representing lipopolysaccharides increases its value. In this case, inflammation doesn’t suffer any change. Contrarily, if we modify the lipopolysaccharides value altogether with astaxanthin, anthocyanin, lycopene, punicalagin (suppressing their values), this condition promotes the NF-κB and M1 macrophages recruitment increase (**Figures 7a, b**).

Although the effect of the lipopolysaccharides increase without a decrease in the astaxanthin, anthocyanin, lycopene and punicalagin compounds does not affect the NFκ-B level, only increases the M1 macrophages recruitment; the increase of lipopolysaccharides while we reduce the astaxanthin, anthocyanin, lycopene and punicalagin levels produce a rise in NFκ-B in addition to the M1 macrophages recruitment.

### 3.3 Overall transitions

We resume the responsible critical nodes having the potential to induce a transition from obesity to health (**Table 1**) and from health to disease (**Table 2**).

**Table 1:** Critical nodes that induce a transition from disease to health.

Node	Disease	Health	Action^1^
Anthocyanin and punicalagin			Decrease of NFκ-B and increase in mitochondrial biogenesis
NRG4			Little decrease of NFκ-B and increase in mitochondrial biogenesis
Oleanolic acid			Marked increase in M2 macrophages recruitment, increase in mitochondrial biogenesis and little decrease in NFκ-B

Green cells correspond to node´s values greater than red ones (for example, anthocyanin and punicalagin in the first row increased their values, with the consequence of transit from disease to health. This condition promotes a decrease in NFκ-B and an increase in mitochondrial biogenesis.

NRG4, neuroreguline 4; NFκ-B, nuclear Factor kappa B.

**Table 2:** Critical nodes that induce a transition from health to disease.

Node	Health	Disease	Action
LPS			Increase in M1 macrophages recruitment
Astaxantin, anthocyanin, lycopene, punicalagin			NFκ-B and M1 macrophages recruitment increase

Green cells correspond to node´s values greater than red ones (for example, lipopolysaccharides in the first row increased their values, with the consequence of transit from health to disease. This condition promotes an increase in M1 macrophages recruitment.

LPS, Lipopolysaccharides; NF-κB, nuclear Factor kappa B.

## 4 Discussion

Insulin resistance is a complex disease and a risk factor for the development of cardiometabolic diseases. The molecular mechanisms of insulin resistance in adipose tissue have been widely elucidated and used as a therapeutic target. The research of pharmacological interventions with the use of bioactive compounds also has been widely investigates with some molecular targets chosen for detailed analysis. However, the use of mathematical networks provides a tool for organizing large amounts of data in nodes to identify modules linked to a specific target that are reliably active only in the context of IR progression.

The study with mathematical models of the interactions (nodes and subnetworks) occurring between chronic-degenerative and infectious diseases with environmental, genetic and molecular factors (macromolecular complexes, enzymes, translation factors and genetic transcription) and, specifically, bioactive compounds or nutraceuticals, have been widely explored in recent decades by the global scientific community (71–74). These investigations have allowed us to understand the pathophysiological manifestations of diseases in different study models and the behavior of pharmacological and nutritional interventions in different pathologies, which can allow the construction of guides focused on new non-pharmacological treatments and the improvement or prevention in the evolution nature of chronic diseases (75–77). This has been widely reported in recent years, particularly in viral diseases, where the involvement and prediction of viral proteins, certain RNA strains, gene mutations or other transcription factors can promote disorder because of their connection and influence in turning on part of the system of these complex networks, and even identifying the initial cellular origin of the infectivity of viruses, providing significant information on the complex structures of interactions in these diseases (78–80).

We present a network constructed between obesity and different risk factors, based on the interactions of mitochondrial biogenesis, inflammatory pathways, insulin signaling and the expression of type 1 and 2 macrophages as well as different bioactive compounds. It has been reported that each of these elements could modulate or act as regulatory factors in adipose tissue.

Our analysis showed that the metabolic pathways commonly linked to obesity and insulin resistance (inflammation, oxidative stress, dysfunctional mitochondrial, etc.) exhibit a synergism with both extrinsic and intrinsic factors in a healthy and diseased state, such as anthocyanins and punicalagin. A possible explanation is that anthocyanins and punicalagin are a type of polyphenolic compounds with antioxidant and anti-inflammatory properties. Numerous pre-clinical and clinical studies have indicated that these polyphenolic compounds have significant efficacy in the prevention of malignant tumors, diabetes mellitus and cardiovascular diseases (81–84). A main source of these bioactive compounds is found in fresh foods such as blueberries, pomegranate, and cherries.

In order to know about the interdependence between adipose tissue response under insulin resistance and the dynamics in the network, we performed a simulation of all nodes associated with bioactive compounds reported to have an essential biological activity. Our analysis found that, when passing from an obesogenic to a healthy state, the main nodes capable of modulating the system by decreasing inflammatory processes regulated by cytokines and macrophages type 1, besides lipid homeostasis, were bioactive compounds such as anthocyanin and punicalagin, likewise oleanolic acid, and to adipokine, NRG4. Our results showed NRG4 as a main node that increases mitochondrial biogenesis, while not reduce inflammation drastically. However, altogether with oleanolic acid, the reduction of NF-κB is evident. On the other hand, when simulating from healthy to obesogenic state, significant changes in the network were the increase of NF-κB and the recruitment of type 1 macrophages with a shutdown of the nodes corresponding to bioactive compounds, whereas the critical node after the total resolutions in the system were the lipopolysaccharides.

Our mathematical model showed a discernment between the impact of various bioactive substances on the transitions from health to obesity and vice versa. In this context, of all the bioactive substances reported in our bibliographic research, our results established a hierarchy of importance between nodes to make a transition between final states associated with health or disease. We found that anthocyanins, punicalagin, oleanolic acid and NRG4 proved to be critical nodes in the transition from obesity to the healthy state, due to their individual switch-on potential to up-regulate the complex network resulting in beneficial transition. Historically, these nodes have been described in pre-clinical and clinical studies as anti-inflammatory, with antioxidant properties and positively influencing PPAR- γ pathway enhancing insulin response (85–87). There is evidence suggesting that oleanolic acid may improve inflammation and oxidative damage caused by diabetes (88, 89) since it improves the response to insulin (90). Additionally, anticancer, anti-inflammatory and antiatherogenic activities have been attributed to this molecule. In this context, *in vitro* studies with HepG2 cells, oleanolic acid can reduce lipid accumulation; a possible mechanism is through the negative regulation of gene transcription and protein expression of PPAR-γ, C/EBP-β and Sterol regulatory element-binding protein-1 (SREBP-1c). A crucial factor in regulating adipocyte differentiation, lipogenesis, and glucose metabolism gene expression is PPAR-γ (91). Likewise, SREBP1-C is an activator of PPAR-γ production and controls the expression of acid synthase. CCAAT-enhancing/binding protein β (C/EBPβ) plays a role in the induction of PPAR-γ and SREBP1-C. As a regulator of early adipocytes, as well as in maintaining the expression level in mature adipocytes. NRG4, is an adipokine, a secreted factor by brown adipose tissue, and its signaling protects hepatocytes from stress-induced injuries and attenuates those induced by a high-fat diet. Reduced NRG4 expression in adipose tissue and plasma levels has been associated with obesity and insulin resistance (92–95). NRG4 signaling has been implicated in the regulation of macrophage survival and function during intestinal inflammation (96, 97). Also, BAT-specific NRG4 deficiency accelerates vascular inflammation and adhesion responses, endothelial dysfunction and apoptosis and atherosclerosis in male mice.

On the other hand, another critical node from healthy to obesogenic state was LPS. In this context, bacterial endotoxins/LPS contributes to acute and chronic inflammations and triggers the innate immune response characterized by cytokine release and immune system activation. Changes in gut microbiota (a common condition in obesity) may induce endotoxemia processes produced by dysbiosis or disruption of the intestinal epithelial barrier which are important determinants involved in the pathophysiology of obesity in inflammatory processes and insulin resistance (98–100).

Network regulatory relations generate a cellular dynamic that portrays disease progression as a transit between attractors corresponding to health and obesity. The importance of the model used here was the identification of key nodes that have the potential to drive the final condition of obesity to a health state and vice versa. Findings of this study are limited by the adipocyte network design considered here, where we included inflammation pathways and inhibition of insulin signaling, insulin signaling and GLUT 4 translocation, triglycerides production, ATP production, M2 macrophages recruitment, adipogenesis and lipolysis, mitochondrial biogenesis and bioactive compounds. A mathematical model is never a complete representation of a biological system, even if it is complex; it is limited to identifying which component or components are responsible for a particular behavior. Therefore, one of the limitations of mathematical models is their reproducibility on a large scale. It is therefore crucial to have a biological system that allows us to investigate the predictions generated by this mathematical model.

One of the limitations of mathematical models is their large-scale reproducibility. Therefore, it is crucial to have a biological system that allows us to investigate the predictions generated by this mathematical model. In this sense, this network has an important role to elucidate or predict the activity of pathway endpoints (phenotypes) (101), drug targets (102) and crosstalk (103). The suggested application of the results of this network is the use of the nodes as potential targets for the design of new pharmacological strategies to transition from obesity to health. In the study design, the researcher should evaluate the M2/M1 macrophages recruitment and NF-kB expression.

Other pharmacological strategies could include the combination of anthocyanins with punicalagin to reduce proinflammatory states, measuring NF-κB in obesity models. In addition, the use of astaxanthin, anthocyanin, lycopene, and punicalagin should be investigated as a pretreatment to avoid the development of insulin resistance via LPS and M1 macrophages recruitment. However, this network may be complemented by inclusion of supplementary elements that may provide a more detailed description of the cellular mechanisms leading to obesity or an improved state of health.

## 5 Conclusion

This study uses a complex network composed of critical nodes which have been reported to interact both directly and indirectly during an obesogenic and insulin resistance process in order to select the nodes most important for the transition between a disease state and a healthy one and vice versa. Using this predictive mathematical model, it is possible to target treatments or molecular mechanisms in a short period of time. Controlled studies with the reported nodes are necessary to assess the effectiveness of the complex network and mathematical simulations.

## Data Availability

The raw data supporting the conclusions of this article will be made available by the authors, without undue reservation.

## Supplementary material

Supplementary Table 1 We summarize the information of the literature review; for every node, we associated it with a value between zero and one for a state of health and obesity. Green cells correspond to node’s values greater than red ones (for example, anthocyanin has a higher expression level in health than in obesity). ^1^ The column health relates the initial values of every node in the case of health ^2^ The column obesity shows the common values in each node compared to the healthy state. HSL, hormone-sensitive lipase; NRG4, neuroreguline 4; VEGFα, vascular endothelial growth factor alpha; ChREBP-B, carbohydrate-responsive element-binding protein beta; Akt, protein kinase B; GLUT4, glucose transporter 4; ATP, adenosin triphosphate; TLR2/4, Toll-like receptor 2/4; NF-κB, nuclear Factor-kappaB; TNFα, tumor necrosis factor alpha STAT3, signal transducer and activator of transcription 3; SOCS3, suppressor of cytokine signaling 3; STAT1, signal transducer and activator of transcription 1; IL-6, interleukine 6; Th1, helper T cell 1; AP-1, AP-1, activator protein-1; C/EBPβ, CCAAT/enhancer‐binding protein beta; C/EBPα, CCAAT/enhancer‐binding protein alpha; PPAR-γ, peroxisome proliferator-activated receptor gamma; IL-4, interleukine 4; pSTAT6, phosphorylated signal transducer and activator of transcription 6; IL-10, interleukine 10; AMPK, AMP-activated protein kinase; SIRT1, silent information regulator sirtuin 1; pPGC1, phosphorylated peroxisome proliferator-activated receptor gamma coactivator.
