# Supplementary material for: Complex networks interactions between bioactive compounds and adipose tissue vis-à-vis insulin resistance
Source: Front Endocrinol (Lausanne). 2025 May 13;16:1578552. doi: 10.3389/fendo.2025.1578552 (PMC12106009; doi:10.3389/fendo.2025.1578552)
Supplement: Supplementary Table 1 — We summarize the information of the literature review; for every node, we associated it with a value between zero and one for a state of health and obesity. Green cells correspond to node’s values greater than red ones (for example, anthocyanin has a higher expression level in health than in obesity). 1 The column health relates the initial values of every node in the case of health 2 The column obesity shows the common values in each node compared to the healthy state. HSL, hormone-sensitive lipase; NRG4, neuroreguline 4; VEGFα, vascular endothelial growth factor alpha; ChREBP-B, carbohydrate-responsive element-binding protein beta; Akt, protein kinase B; GLUT4, glucose transporter 4; ATP, adenosin triphosphate; TLR2/4, Toll-like receptor 2/4; NF-κB, nuclear Factor-kappaB; TNFα, tumor necrosis factor alpha STAT3, signal transducer and activator of transcription 3; SOCS3, suppressor of cytokine signaling 3; STAT1, signal transducer and activator of transcription 1; IL-6, interleukine 6; Th1, helper T cell 1; AP-1, AP-1, activator protein-1; C/EBPβ, CCAAT/enhancer‐binding protein beta; C/EBPα, CCAAT/enhancer‐binding protein alpha; PPAR-γ, peroxisome proliferator-activated receptor gamma; IL-4, interleukine 4; pSTAT6, phosphorylated signal transducer and activator of transcription 6; IL-10, interleukine 10; AMPK, AMP-activated protein kinase; SIRT1, silent information regulator sirtuin 1; pPGC1, phosphorylated peroxisome proliferator-activated receptor gamma coactivator. [file DataSheet1.pdf]

Supplementary Material

Node	Health[1]	Obesity[2]	Cite
Anthocyanin			[52]
Cyanidin-3-o-β			[58]
Astaxantin			[59]
Lycopene			[59]
Punicalagin			[50]
HSL			[41]
Capsaicin			[43]
Resveratrol			[46]
Bilobalide			[41]
Berberine			[45]
Isoorientin			[44]
Oleanolic acid			[51]
NRG4			[49]
Nobilentin			[60]
Celastron			[53]
Pyrroloquinoline quinone			[42]
Allicin			[49]

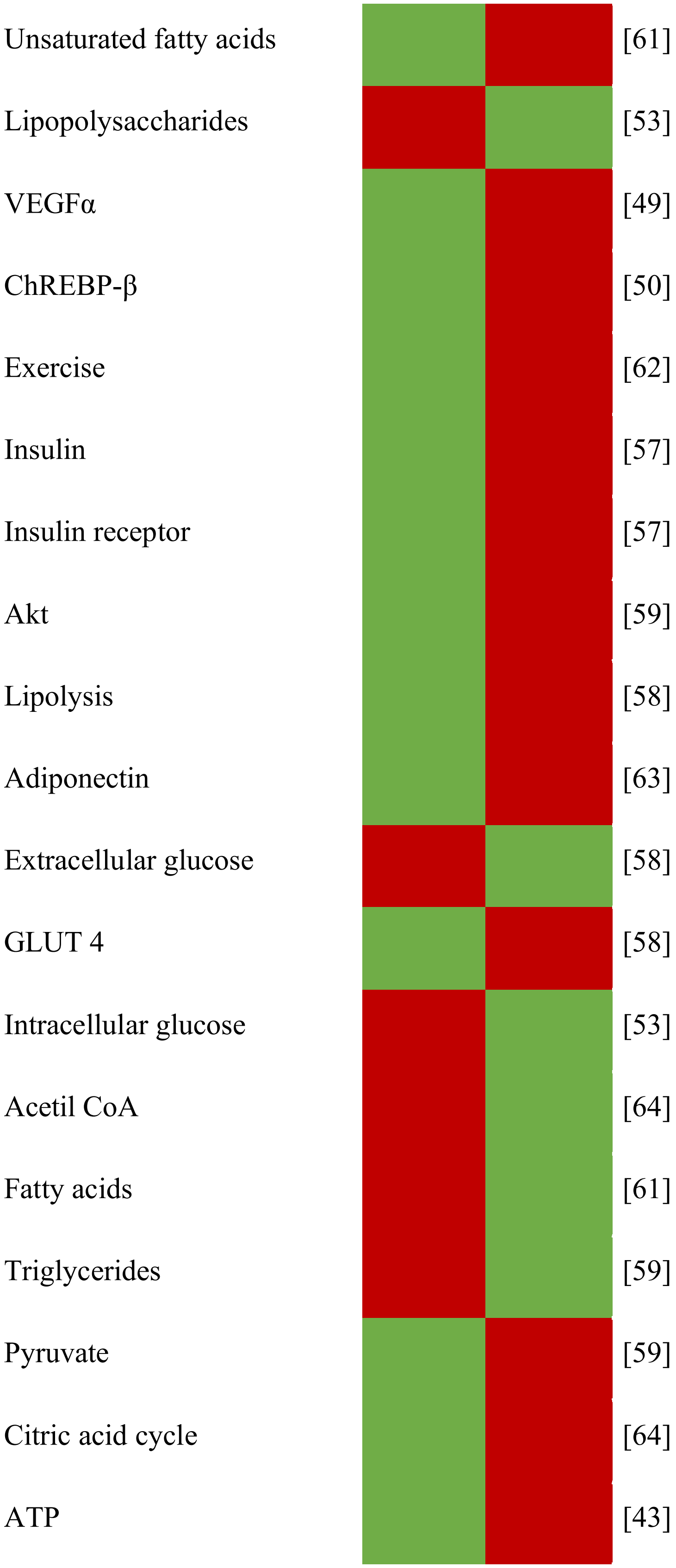
Unsaturated fatty acids [61]
Lipopolysaccharides [53]
VEGFα [49]
ChREBP-β [50]
Exercise [62]
Insulin [57]
Insulin receptor [57]
Akt [59]
Lipolysis [58]
Adiponectin [63]
Extracellular glucose [58]
GLUT 4 [58]
Intracellular glucose [53]
Acetil CoA [64]
Fatty acids [61]
Triglycerides [59]
Pyruvate [59]
Citric acid cycle [64]
ATP [43]

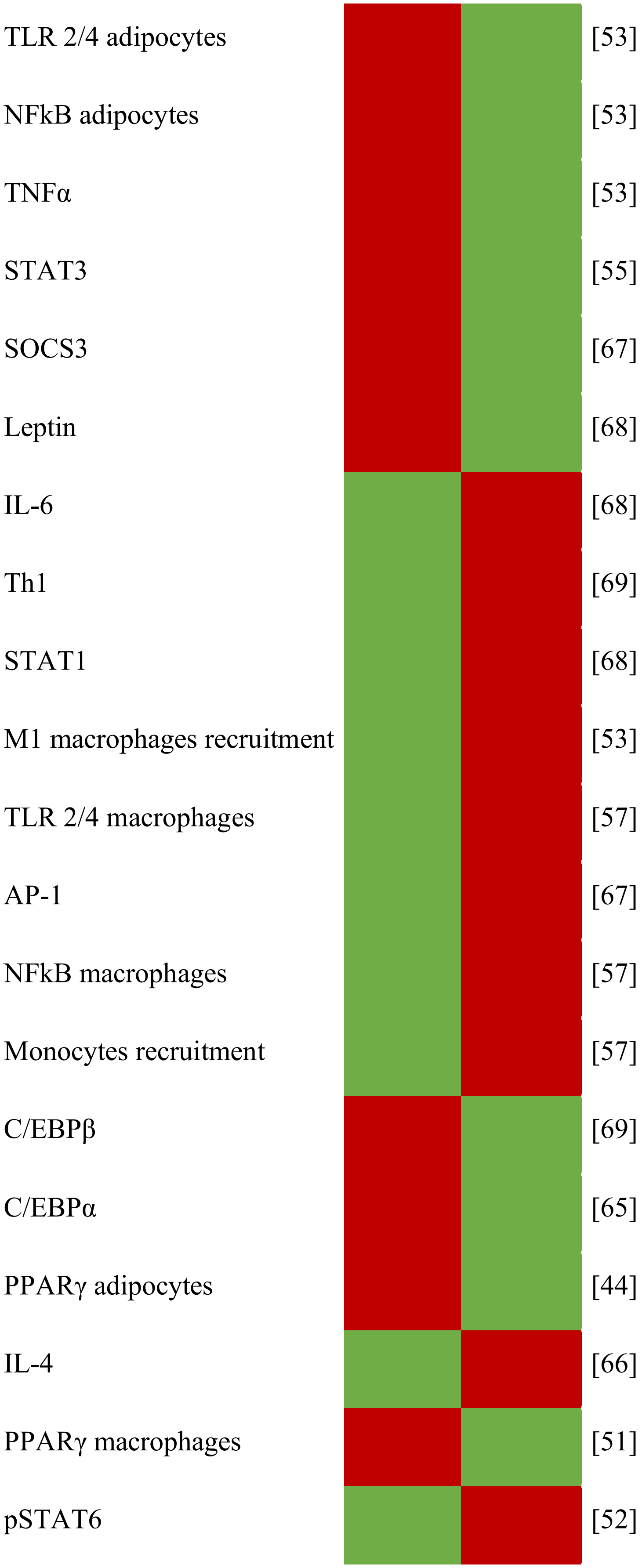
TLR 2/4 adipocytes [53]
NFkB adipocytes [53]
TNFα [53]
STAT3 [55]
SOCS3 [67]
Leptin [68]
IL-6 [68]
Th1 [69]
STAT1 [68]
M1 macrophages recruitment [53]
TLR 2/4 macrophages [57]
AP-1 [67]
NFkB macrophages [57]
Monocytes recruitment [57]
C/EBPβ [69]
C/EBPα [65]
PPARγ adipocytes [44]
IL-4 [66]
PPARγ macrophages [51]
pSTAT6 [52]

M2 macrophages recruitment			[50]
IL-10			[66]
AMPK			[49]
SIRT1			[45]
pPGC1			[48]
Mitochondrial biogenesis			[47]

Table 2S. We summarize the information of the literature review; for every node, we associated it with a value between zero and one for a state of health and obesity. Green cells correspond to node's values greater than red ones (for example, anthocyanin has a higher expression level in health than in obesity).

[1] The column health relates the initial values of every node in the case of health

[2] The column obesity shows the common values in each node compared to the healthy state.

HSL: hormone-sensitive lipase; NRG4: neuroreguline 4; VEGFα: vascular endothelial growth factor alpha; ChREBP-B: carbohydrate-responsive element-binding protein beta; Akt: protein kinase B; GLUT4: glucose transporter 4; ATP: adenosin triphosphate; TLR2/4: Toll-like receptor 2/4; NF-κB: nuclear Factor-kappaB; TNFα: tumor necrosis factor alpha STAT3: signal transducer and activator of transcription 3; SOCS3: suppressor of cytokine signaling 3; STAT1: signal transducer and activator of transcription 1; IL-6: interleukine 6; Th1: helper T cell 1; AP-1: AP-1: activator protein-1; C/EBPβ: CCAAT/enhancer-binding protein beta; C/EBPα: CCAAT/enhancer-binding protein alpha; PPARγ: peroxisome proliferator-activated receptor gamma; IL-4: interleukine 4; pSTAT6: phosphorylated signal transducer and activator of transcription 6; IL-10: interleukine 10; AMPK: AMP-activated protein kinase; SIRT1: silent information regulator sirtuin 1; pPGC1: phosphorylated peroxisome proliferator-activated receptor gamma coactivator.

Explanation Supplementary 1

We used Wolfram Mathematica software to solve the ordinary differential equations describing the expression pattern in each health- or disease-state and the transitions between them. Initially, we established the fuzzy logic propositions for all nodes, then we defined the decay rates ($b=5$) and the saturation parameter ($\alpha = 1$) for each node. Every attractor was considered in the next step as a set of initial conditions of each node and with the previous initial conditions we solved the equations, showing the results as graphs. For more detail about the mathematical description and parameters, see the Material and methods section in the main text.

In[ ]:=

```
winsr[t_] := insulin[t]
wnfkbad[t_] := lipopoly[t] (1 - (punical[t] + lycop[t] - punical[t] × lycop[t]))
   (1 - (astax[t] + anthocy[t] - astax[t] × anthocy[t]))
wstatuno[t_] := thuno[t]
wtlrad[t_] := lipopoly[t]
wtlrmac[t_] := lipopoly[t]
wapuno[t_] := lipopoly[t] + lipopoly[t] - lipopoly[t] × lipopoly[t]
wnfkbmac[t_] :=
  (celastr[t] + lipopoly[t] - celastr[t] × lipopoly[t]) (1 - (unsatfatac[t]))
wmonocy[t_] := (lipopoly[t] + lipopoly[t] - lipopoly[t] × lipopoly[t]) +
   (celastr[t] + lipopoly[t] - celastr[t] × lipopoly[t]) (1 - (unsatfatac[t])) -
   (lipopoly[t] + lipopoly[t] - lipopoly[t] × lipopoly[t])
    ((celastr[t] + lipopoly[t] - celastr[t] × lipopoly[t]) (1 - (unsatfatac[t])))
wildiez[t_] := ilcuatro[t]
wmdosmacrop[t_] := (ilcuatro[t] + oleanacid[t]) - (ilcuatro[t] × oleanacid[t])
wstatseis[t_] := ilcuatro[t]
wppargm[t_] := ilcuatro[t]
   (1 - ((celastr[t] + lipopoly[t] - celastr[t] × lipopoly[t]) (1 - (unsatfatac[t])) +
       oleanacid[t] - ((celastr[t] + lipopoly[t] - celastr[t] × lipopoly[t])
          (1 - (unsatfatac[t])) oleanacid[t])))
wpparga[t_] := (cebpb[t] + isoorien[t] - cebpb[t] × isoorien[t])
   (1 - (punical[t] + (lipopoly[t] (1 - (punical[t] + lycop[t] - punical[t] × lycop[t]))
          (1 - (astax[t] + anthocy[t] - astax[t] × anthocy[t]))) -
       punical[t] (lipopoly[t] (1 - (punical[t] + lycop[t] - punical[t] × lycop[t]))
           (1 - (astax[t] + anthocy[t] - astax[t] × anthocy[t]))))) (1 - (oleanacid[t]))
wcebpa[t_] := cebpb[t] + (cebpb[t] + isoorien[t] - cebpb[t] × isoorien[t])
    (1 - (punical[t] + (lipopoly[t] (1 - (punical[t] + lycop[t] - punical[t] × lycop[t]))
           (1 - (astax[t] + anthocy[t] - astax[t] × anthocy[t]))) -
         punical[t] (lipopoly[t] (1 - (punical[t] + lycop[t] - punical[t] × lycop[t]))
             (1 - (astax[t] + anthocy[t] - astax[t] × anthocy[t]))))) (1 - (oleanacid[t])) -
   cebpb[t] ((cebpb[t] + isoorien[t] - cebpb[t] × isoorien[t])
       (1 - (punical[t] + (lipopoly[t] (1 - (punical[t] + lycop[t] - punical[t] × lycop[t]))
              (1 - (astax[t] + anthocy[t] - astax[t] × anthocy[t]))) -
           punical[t] (lipopoly[t] (1 - (punical[t] + lycop[t] - punical[t] × lycop[t])) (1 -
                  (astax[t] + anthocy[t] - astax[t] × anthocy[t]))))) (1 - (oleanacid[t])))
wglut[t_] := (pakt[t] + chre[t] - pakt[t] × chre[t] + extrag[t] + nrg[t] - extrag[t] × nrg[t] -
      (pakt[t] + chre[t] - pakt[t] × chre[t]) (extrag[t] + nrg[t] - extrag[t] × nrg[t])) +
   celastr[t] - (pakt[t] + chre[t] - pakt[t] × chre[t] + extrag[t] + nrg[t] -
      extrag[t] × nrg[t] - (pakt[t] + chre[t] - pakt[t] × chre[t])
        (extrag[t] + nrg[t] - extrag[t] × nrg[t])) celastr[t]
wlipol[t_] := ((cyanidin[t] + oleanacid[t] - cyanidin[t] × oleanacid[t]) +
     hsl[t] - (cyanidin[t] + oleanacid[t] - cyanidin[t] × oleanacid[t]) hsl[t])
   (1 - (pakt[t] + pparga[t] - pakt[t] × pparga[t]))
whsl[t_] := bilobalide[t] (1 - (punical[t]))
wadiponectin[t_] := (anthocy[t] + nrg[t] - anthocy[t] × nrg[t]) (1 - (lipopoly[t]))
```

```
wintrag[t_] := glut[t]
wpiruvate[t_] := glut[t]
wcitric[t_] := glut[t]
watp[t_] := glut[t]
wacetil[t_] := glut[t]
wfattyac[t_] := glut[t]
wtrigly[t_] := glut[t]
wmuno[t_] := statres[t] + statuno[t] - statres[t] × statuno[t] +
  monocy[t] - (statres[t] + statuno[t] - statres[t] × statuno[t]) monocy[t]
wchre[t_] := anthocy[t]
wtnf[t_] := muno[t] (1 - punical[t]) (1 - anthocy[t])
wsocs[t_] := statres[t]
winsulin[t_] := insulin[t]
wlipopoly[t_] := lipopoly[t]
wpunical[t_] := punical[t]
wlycop[t_] := lycop[t]
wastax[t_] := astax[t]
wanthocy[t_] := anthocy[t]
wthuno[t_] := thuno[t]
wcelastr[t_] := celastr[t]
wunsatfatac[t_] := unsatfatac[t]
wilcuatro[t_] := ilcuatro[t]
woleanacid[t_] := oleanacid[t]
wcebpb[t_] := cebpb[t]
wisoorien[t_] := isoorien[t]
wnrg[t_] := nrg[t]
wpakt[t_] := pakt[t]
wextrag[t_] := extrag[t]
wcyanidin[t_] := cyanidin[t]
wbilobalide[t_] := bilobalide[t]
wstatres[t_] := statres[t]
wexercise[t_] := exercise[t]
wcapsaicin[t_] := capsaicin[t]
wresveratrol[t_] := resveratrol[t]
wberberine[t_] := berberine[t]
wallicin[t_] := allicin[t]
wpyrro[t_] := pyrro[t]
wnobi[t_] := nobi[t]
wveg[t_] := nrg[t]
wleptin[t_] := leptin[t]
wampk[t_] := ((adiponectin[t] + isoorien[t] - adiponectin[t] × isoorien[t]) +
    (capsaicin[t] + resveratrol[t] - capsaicin[t] × resveratrol[t]) -
    (adiponectin[t] + isoorien[t] - adiponectin[t] × isoorien[t])
     (capsaicin[t] + resveratrol[t] - capsaicin[t] × resveratrol[t]) +
    (bilobalide[t] + exercise[t] - bilobalide[t] × exercise[t]) +
    (berberine[t] + sirt[t] - berberine[t] × sirt[t]) -
    (bilobalide[t] + exercise[t] - bilobalide[t] × exercise[t])
     (berberine[t] + sirt[t] - berberine[t] × sirt[t])
    - (((adiponectin[t] + isoorien[t] - adiponectin[t] × isoorien[t]) +
          (capsaicin[t] + resveratrol[t] - capsaicin[t] × resveratrol[t]) -
```

```
          (adiponectin[t] + isoorien[t] - adiponectin[t] × isoorien[t])
           (capsaicin[t] + resveratrol[t] - capsaicin[t] × resveratrol[t])) -
        (adiponectin[t] + isoorien[t] - adiponectin[t] × isoorien[t])
         (capsaicin[t] + resveratrol[t] - capsaicin[t] × resveratrol[t]))
      ×
      ((bilobalide[t] + exercise[t] - bilobalide[t] × exercise[t]) +
        (berberine[t] + sirt[t] - berberine[t] × sirt[t]) -
        (bilobalide[t] + exercise[t] - bilobalide[t] × exercise[t])
         (berberine[t] + sirt[t] - berberine[t] × sirt[t])))
(1 - atp[t])
wsirt[t_] := ampk[t] + resveratrol[t] - ampk[t] × resveratrol[t] +
  berberine[t] - (ampk[t] + resveratrol[t] - ampk[t] × resveratrol[t]) berberine[t]
wmitoc[t_] := pparga[t] + pyrro[t] - pparga[t] × pyrro[t] + celastr[t] -
  (pparga[t] + pyrro[t] - pparga[t] × pyrro[t]) celastr[t] + allicin[t] -
  (pparga[t] + pyrro[t] - pparga[t] × pyrro[t] + celastr[t] -
     (pparga[t] + pyrro[t] - pparga[t] × pyrro[t]) celastr[t]) allicin[t] +
  pgc[t] - (pparga[t] + pyrro[t] - pparga[t] × pyrro[t] + celastr[t] -
     (pparga[t] + pyrro[t] - pparga[t] × pyrro[t]) celastr[t] + allicin[t] -
     (pparga[t] + pyrro[t] - pparga[t] × pyrro[t] + celastr[t] -
        (pparga[t] + pyrro[t] - pparga[t] × pyrro[t]) celastr[t]) allicin[t]) pgc[t]
wpgc[t_] := sirt[t]
wilseis[t_] := muno[t] (1 - anthocy[t]) (1 - punical[t])
```

Out[•]= 1 - atp[t]

In[•]:=

In[•]:=

In[•]:=
```
b = 5.5;
dinsr = 1;
dinsulin = 1;
dnfkbad = 1;
dstatuno = 1;
dtlrad = 1;
dtlrmac = 1;
dapuno = 1;
dlipopoly = 1;
dpunical = 1;
dlycop = 1;
dastax = 1;
danthocy = 1;
dthuno = 1;
dnfkbmac = 1;
dcelastr = 1;
dunsatfatac = 1;
dmonocy = 1;
dildiez = 1;
```

```
dilcuatro = 1;
dmdosmacrop = 1;
dstatseis = 1;
dppargm = 1;
dpparga = 1;
doleanacid = 1;
dcebpb = 1;
disoorien = 1;
dcebpa = 1;
dglut = 1;
dnrg = 1;
dpakt = 1;
dextrag = 1;
dlipol = 1;
dhsl = 1;
dcyanidin = 1;
dbilobalide = 1;
dadiponectin = 1;
dintrag = 1;
dpiruvate = 1;
dcitric = 1;
datp = 1;
dacetil = 1;
dfattyac = 1;
dtrigly = 1;
dmuno = 1;
dstatres = 2;
dexercise = 1;
dcapsaicin = 1;
dresveratrol = 1;
dberberine = 1;
dallicin = 1;
dpyrro = 1;
dnobi = 1;
dveg = 1;
dchre = 1;
dtnf = 1;
dleptin = 1;
dsocs = 1;
dampk = 1;
dsirt = 1;
dmitoc = 1;
dpgc = 1;
dilseis = 1;
```

In[ ]:=

```
insr0 = 0.5;
insulin0 = 0.5;
nfkbad0 = 1;
statuno0 = 1;
```

```
tlrad0 = 2;
tlrmac0 = 1;
apuno0 = 1;
lipopoly0 = 2;
punical0 = 1;
lycop0 = 0;
astax0 = 0;
anthocy0 = 1;
thuno0 = 1;
nfkbmac0 = 1;
celastr0 = 0;
unsatfatac0 = 0;
monocy0 = 1;
ildiez0 = 1;
ilcuatro0 = 0;
mdosmacrop0 = 0;
statseis0 = 0;
ppargm0 = 0.5;
pparga0 = 0.5;
oleanacid0 = 0;
cebpb0 = 0.5;
isoorien0 = 0;
cebpa0 = 0.5;
glut0 = 0.5;
nrg0 = 0;
pakt0 = 1;
extrag0 = 1;
lipol0 = 0.5;
hsl0 = 0;
cyanidin0 = 0;
bilobalide0 = 0;
adiponectin0 = 0;
intrag0 = 1;
piruvate0 = 1;
citric0 = 1;
atp0 = 1;
acetil0 = 1;
fattyac0 = 1;
trigly0 = 1;
muno0 = 1;
statres0 = 1;
exercise0 = 0;
capsaicin0 = 0;
resveratrol0 = 0;
berberine0 = 0;
allicin0 = 0;
pyrro0 = 0;
nobi0 = 0;
veg0 = 0;
chre0 = 0;
tnf0 = 1;
```

```
leptin0 = 0.5;
socs0 = 1;
ampk0 = 0.5;
sirt0 = 0;
mitoc0 = 0.5;
pgc0 = 0;
ilseis0 = 1;
```

In[ ]:=
```
betahepato = NDSolve[{
                resolvedor diferencial numérico
    insr'[t] == (1/(1 + Exp[-b (winsr[t] - .5)]) - dinsr insr[t]),
                        exponencial
    insulin'[t] == (1/(1 + Exp[-b (winsulin[t] - .5)]) - dinsulin insulin[t]),
                        exponencial
    nfkbad'[t] == (1/(1 + Exp[-b (wnfkbad[t] - .5)]) - dnfkbad nfkbad[t]),
                        exponencial
    statuno'[t] == (1/(1 + Exp[-b (wstatuno[t] - .5)]) - dstatuno statuno[t]),
                        exponencial
    tlrad'[t] == (1/(1 + Exp[-b (wtlrad[t] - .5)]) - dtlrad tlrad[t]),
                        exponencial
    tlrmac'[t] == (1/(1 + Exp[-b (wtlrmac[t] - .5)]) - dtlrmac tlrmac[t]),
                        exponencial
    apuno'[t] == (1/(1 + Exp[-b (wapuno[t] - .5)]) - dapuno apuno[t]),
                        exponencial
    lipopoly'[t] == (1/(1 + Exp[-b (wlipopoly[t] - .5)]) - dlipopoly lipopoly[t]),
                        exponencial
    punical'[t] == (1/(1 + Exp[-b (wpunical[t] - .5)]) - dpunical punical[t]),
                        exponencial
    lycop'[t] == (1/(1 + Exp[-b (wlycop[t] - .5)]) - dlycop lycop[t]),
                        exponencial
    astax'[t] == (1/(1 + Exp[-b (wastax[t] - .5)]) - dastax astax[t]),
                        exponencial
    anthocy'[t] == (1/(1 + Exp[-b (wanthocy[t] - .5)]) - danthocy anthocy[t]),
                        exponencial
    thuno'[t] == (1/(1 + Exp[-b (wthuno[t] - .5)]) - dthuno thuno[t]),
                        exponencial
    nfkbmac'[t] == (1/(1 + Exp[-b (wnfkbmac[t] - .5)]) - dnfkbmac nfkbmac[t]),
                        exponencial
    celastr'[t] == (1/(1 + Exp[-b (wcelastr[t] - .5)]) - dcelastr celastr[t]),
                        exponencial
    unsatfatac'[t] == (1/(1 + Exp[-b (wunsatfatac[t] - .5)]) - dunsatfatac unsatfatac[t]),
                        exponencial
    monocy'[t] == (1/(1 + Exp[-b (wmonocy[t] - .5)]) - dmonocy monocy[t]),
                        exponencial
    ildiez'[t] == (1/(1 + Exp[-b (wildiez[t] - .5)]) - dildiez ildiez[t]),
                        exponencial
    ilcuatro'[t] == (1/(1 + Exp[-b (wilcuatro[t] - .5)]) - dilcuatro ilcuatro[t]),
                        exponencial
```

```
exponencial
mdosmacrop'[t] == (1/(1 + Exp[-b (wmdosmacrop[t] - .5)]) - dmdosmacrop mdosmacrop[t]),
exponencial
statseis'[t] == (1/(1 + Exp[-b (wstatseis[t] - .5)]) - dstatseis statseis[t]),
exponencial
ppargm'[t] == (1/(1 + Exp[-b (wppargm[t] - .5)]) - dppargm ppargm[t]),
exponencial
pparga'[t] == (1/(1 + Exp[-b (wpparga[t] - .5)]) - dpparga pparga[t]),
exponencial
oleanacid'[t] == (1/(1 + Exp[-b (woleanacid[t] - .5)]) - doleanacid oleanacid[t]),
exponencial
cebpb'[t] == (1/(1 + Exp[-b (wcebpb[t] - .5)]) - dcebpb cebpb[t]),
exponencial
isoorien'[t] == (1/(1 + Exp[-b (wisoorien[t] - .5)]) - disoorien isoorien[t]),
exponencial
cebpa'[t] == (1/(1 + Exp[-b (wcebpa[t] - .5)]) - dcebpa cebpa[t]),
exponencial
glut'[t] == (1/(1 + Exp[-b (wglut[t] - .5)]) - dglut glut[t]),
exponencial
nrg'[t] == (1/(1 + Exp[-b (wnrg[t] - .5)]) - dnrg nrg[t]),
exponencial
pakt'[t] == (1/(1 + Exp[-b (wpakt[t] - .5)]) - dpakt pakt[t]),
exponencial
extrag'[t] == (1/(1 + Exp[-b (wextrag[t] - .5)]) - dextrag extrag[t]),
exponencial
lipol'[t] == (1/(1 + Exp[-b (wlipol[t] - .5)]) - dlipol lipol[t]),
exponencial
hsl'[t] == (1/(1 + Exp[-b (whsl[t] - .5)]) - dhsl hsl[t]),
exponencial
cyanidin'[t] == (1/(1 + Exp[-b (wcyanidin[t] - .5)]) - dcyanidin cyanidin[t]),
exponencial
bilobalide'[t] == (1/(1 + Exp[-b (wbilobalide[t] - .5)]) - dbilobalide bilobalide[t]),
exponencial
adiponectin'[t] ==
  (1/(1 + Exp[-b (wadiponectin[t] - .5)]) - dadiponectin adiponectin[t]),
exponencial
intrag'[t] == (1/(1 + Exp[-b (wintrag[t] - .5)]) - dintrag intrag[t]),
exponencial
piruvate'[t] == (1/(1 + Exp[-b (wpiruvate[t] - .5)]) - dpiruvate piruvate[t]),
exponencial
citric'[t] == (1/(1 + Exp[-b (wcitric[t] - .5)]) - dcitric citric[t]),
exponencial
atp'[t] == (1/(1 + Exp[-b (watp[t] - .5)]) - datp atp[t]),
exponencial
acetil'[t] == (1/(1 + Exp[-b (wacetil[t] - .5)]) - dacetil acetil[t]),
exponencial
fattyac'[t] == (1/(1 + Exp[-b (wfattyac[t] - .5)]) - dfattyac fattyac[t]),
exponencial
```

```
exponencial
trigly'[t] == (1/(1 + Exp[-b (wtrigly[t] - .5)]) - dtrigly trigly[t]),
                  exponencial
muno'[t] == (1/(1 + Exp[-b (wmuno[t] - .5)]) - dmuno muno[t]),
                exponencial
statres'[t] == (1/(1 + Exp[-b (wstatres[t] - .5)]) - dstatres statres[t]),
                  exponencial
exercise'[t] == (1/(1 + Exp[-b (wexercise[t] - .5)]) - dexercise exercise[t]),
                   exponencial
capsaicin'[t] == (1/(1 + Exp[-b (wcapsaicin[t] - .5)]) - dcapsaicin capsaicin[t]),
                    exponencial
resveratrol'[t] ==
  (1/(1 + Exp[-b (wresveratrol[t] - .5)]) - dresveratrol resveratrol[t]),
          exponencial
berberine'[t] == (1/(1 + Exp[-b (wberberine[t] - .5)]) - dberberine berberine[t]),
                    exponencial
allicin'[t] == (1/(1 + Exp[-b (wallicin[t] - .5)]) - dallicin allicin[t]),
                  exponencial
pyrro'[t] == (1/(1 + Exp[-b (wpyrro[t] - .5)]) - dpyrro pyrro[t]),
                exponencial
nobi'[t] == (1/(1 + Exp[-b (wnobi[t] - .5)]) - dnobi nobi[t]),
               exponencial
veg'[t] == (1/(1 + Exp[-b (wveg[t] - .5)]) - dveg veg[t]),
              exponencial
chre'[t] == (1/(1 + Exp[-b (wchre[t] - .5)]) - dchre chre[t]),
               exponencial
tnf'[t] == (1/(1 + Exp[-b (wtnf[t] - .5)]) - dtnf tnf[t]),
              exponencial
leptin'[t] == (1/(1 + Exp[-b (wleptin[t] - .5)]) - dleptin leptin[t]),
                 exponencial
socs'[t] == (1/(1 + Exp[-b (wsocs[t] - .5)]) - dsocs socs[t]),
               exponencial
ampk'[t] == (1/(1 + Exp[-b (wampk[t] - .5)]) - dampk ampk[t]),
               exponencial
sirt'[t] == (1/(1 + Exp[-b (wsirt[t] - .5)]) - dsirt sirt[t]),
               exponencial
mitoc'[t] == (1/(1 + Exp[-b (wmitoc[t] - .5)]) - dmitoc mitoc[t]),
                exponencial
pgc'[t] == (1/(1 + Exp[-b (wpgc[t] - .5)]) - dpgc pgc[t]),
              exponencial
ilseis'[t] == (1/(1 + Exp[-b (wilseis[t] - .5)]) - dilseis ilseis[t]),
                 exponencial
nfkbad[0] == nfkbad0, statuno[0] == statuno0, tlrad[0] == tlrad0, tlrmac[0] == tlrmac0,
apuno[0] == apuno0, lipopoly[0] == lipopoly0, punical[0] == punical0,
lycop[0] == lycop0, astax[0] == astax0, anthocy[0] == anthocy0, thuno[0] == thuno0,
nfkbmac[0] == nfkbmac0, celastr[0] == celastr0, unsatfatac[0] == unsatfatac0,
monocy[0] == monocy0, ildiez[0] == ildiez0, ilcuatro[0] == ilcuatro0,
mdosmacrop[0] == mdosmacrop0, statseis[0] == statseis0, ppargm[0] == ppargm0,
pparga[0] == pparga0, oleanacid[0] == oleanacid0, cebpb[0] == cebpb0,
```

```
    isoorien[0] == isoorien0, cebpa[0] == cebpa0, insr[0] == insr0,
    insulin[0] == insulin0, glut[0] == glut0, nrg[0] == nrg0, pakt[0] == pakt0,
    extrag[0] == extrag0, lipol[0] == lipol0, hsl[0] == hsl0, cyanidin[0] == cyanidin0,
    bilobalide[0] == bilobalide0, adiponectin[0] == adiponectin0, intrag[0] == intrag0,
    piruvate[0] == piruvate0, citric[0] == citric0, atp[0] == atp0,
    acetil[0] == acetil0, fattyac[0] == fattyac0, trigly[0] == trigly0, muno[0] == muno0,
    statres[0] == statres0, exercise[0] == exercise0, capsaicin[0] == capsaicin0,
    resveratrol[0] == resveratrol0, berberine[0] == berberine0, allicin[0] == allicin0,
    pyrro[0] == pyrro0, nobi[0] == nobi0, veg[0] == veg0, chre[0] == chre0,
    tnf[0] == tnf0, leptin[0] == leptin0, socs[0] == socs0, ampk[0] == ampk0,
    sirt[0] == sirt0, mitoc[0] == mitoc0, pgc[0] == pgc0, ilseis[0] == ilseis0},
   {nfkbad[t], statuno[t], tlrad[t], tlrmac[t], apuno[t], lipopoly[t], punical[t],
    lycop[t], astax[t], anthocy[t], thuno[t], nfkbmac[t], celastr[t], unsatfatac[t],
    monocy[t], ildiez[t], ilcuatro[t], mdosmacrop[t], statseis[t], ppargm[t],
    pparga[t], oleanacid[t], cebpb[t], isoorien[t], cebpa[t], insr[t], insulin[t],
    glut[t], nrg[t], pakt[t], extrag[t], lipol[t], hsl[t], cyanidin[t], bilobalide[t],
    adiponectin[t], intrag[t], piruvate[t], citric[t], atp[t], acetil[t], fattyac[t],
    trigly[t], muno[t], statres[t], exercise[t], capsaicin[t], resveratrol[t],
    berberine[t], allicin[t], pyrro[t], nobi[t], veg[t], chre[t], tnf[t],
    leptin[t], socs[t], ampk[t], sirt[t], mitoc[t], pgc[t], ilseis[t]}, {t, 0, 30.}];
```

In[•]:=
```
netw = Plot[Evaluate[{nfkbad[t], mitoc[t], mdosmacrop[t]} /. betahepato], {t, 0, 30},
        repr··· evalúa
   PlotRange → {0, 1}, FrameLabel -> {{Expression level,}, {Time,
   rango de representación  etiqueta de marco  expresión
     "NFKBa(red), mitochondrial biogenesis(orange), M2 macrophages recruitment(black)
"}}, PlotStyle → {{Thick, Red}, {Thick, Orange}, {Thick, Darker[Black]}, {Thick, Pink},
    estilo de represe··· grueso rojo  grueso naranja  grueso más o··· negro  grueso rosa
    {Thick, Purple}, {Thick, Blue}, {Thick, Red}, {Thick, Gray}, {Thick, Black}},
     grueso púrpura  grueso azul  grueso rojo  grueso gris  grueso negro
  Frame → True, PlotLabel → " Disease to health"]
  marco   verd··· etiqueta de representación
```

Out[ ]=

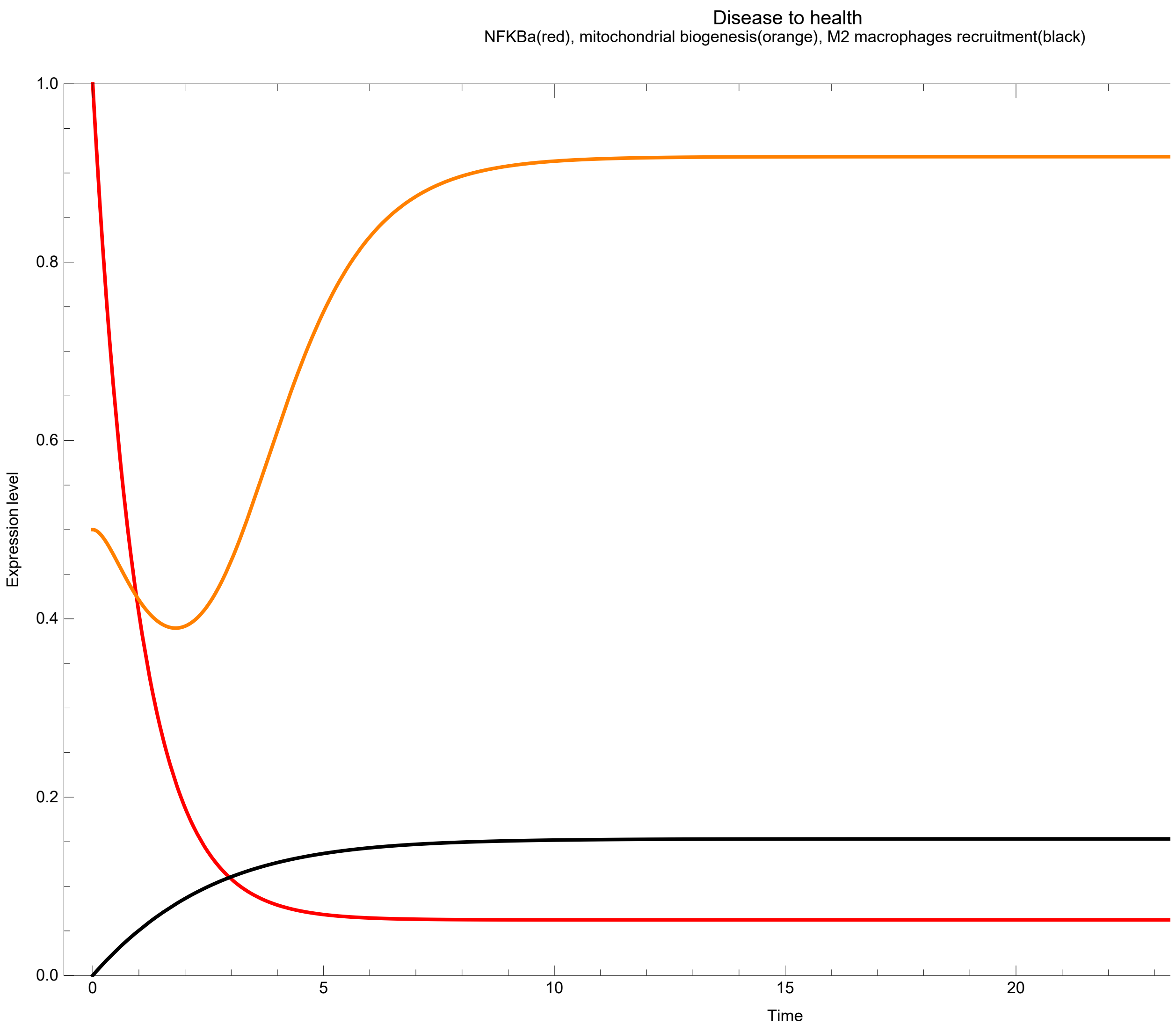
Disease to health
NFKBa(red), mitochondrial biogenesis(orange), M2 macrophages recruitment(black)
Expression level
Time
1.0
0.8
0.6
0.4
0.2
0.0
0
5
10
15
20

In[ ]:=

In[ ]:=
```
netw = Plot[
       representación gráfica
  Evaluate[{muno[t], 0.98 trigly[t], atp[t], 0.94 glut[t]} /. betahepato], {t, 0, 30},
  evalúa
    PlotRange → {0, 1}, FrameLabel -> {{Expression level,}, {Time,
    rango de representación  etiqueta de marco  expresión
      "M1 macrophages recruitment(purple), triglycerides(pink), ATP(green), GLUT4(blue)
"}}, PlotStyle → {{Thick, Purple}, {Thick, Pink}, {Thick, Green}, {Thick, Blue},
     estilo de represe··· grueso púrpura  grueso rosa  grueso verde  grueso azul
    {Thick, Blue}, {Thick, Blue}, {Thick, Red}, {Thick, Gray}, {Thick, Black}},
     grueso azul  grueso azul  grueso rojo  grueso gris  grueso negro
  Frame → True, PlotLabel → " Disease to health"]
  marco  verd··· etiqueta de representación
```

Out[ ]=

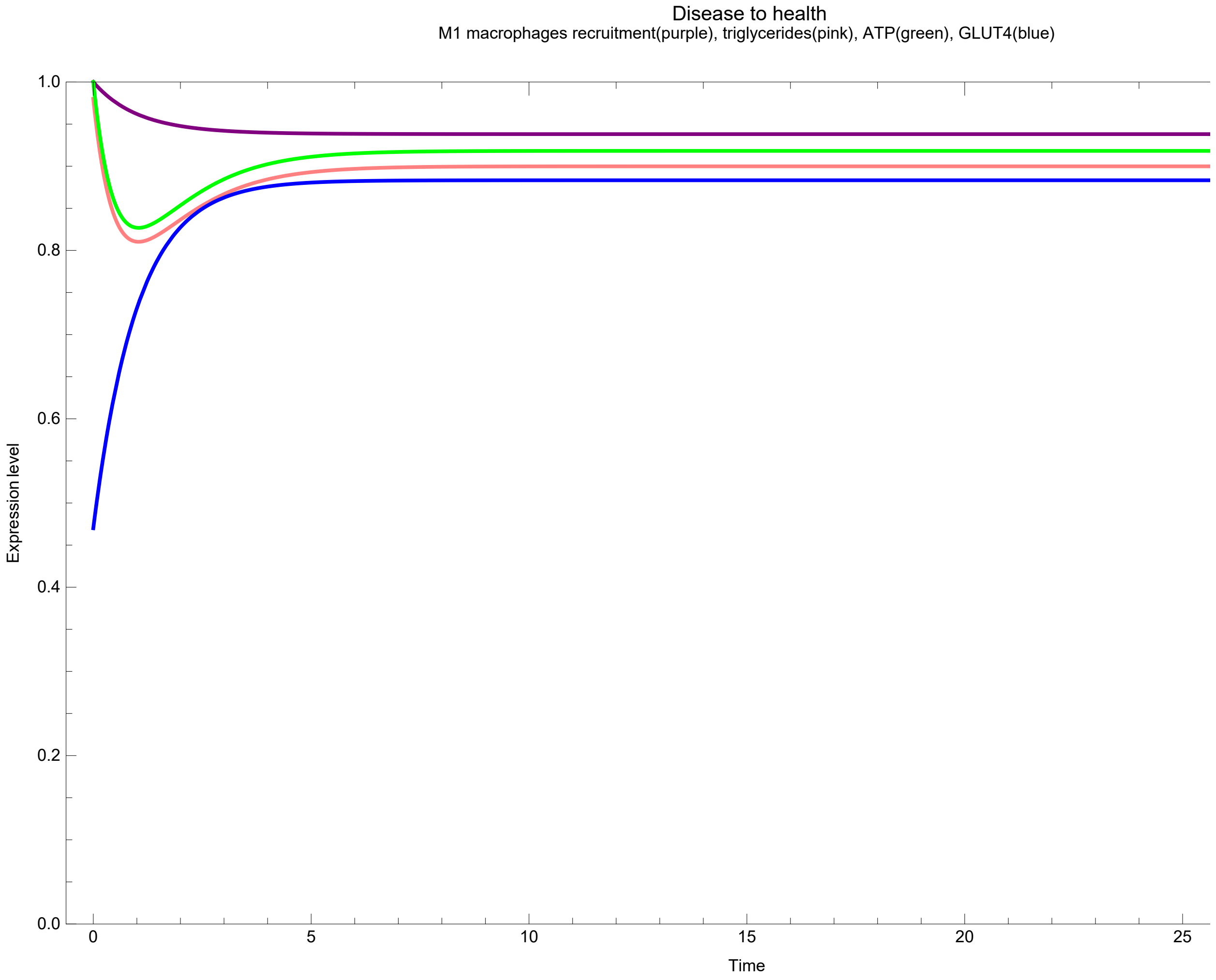

In[ ]:=
